# Assessing content validity: challenges of conducting systematic reviews of patient-reported outcome measures and recommendations to improve the application of COSMIN guidance

**DOI:** 10.1007/s11136-026-04261-5

**Published:** 2026-05-03

**Authors:** Rachel L. Chambers, Silvia Lahuerta-Martín, Ana M. Greco, Rachael Pattinson, Timothy Pickles, Jason Hassan, Lidwine B. Mokkink

**Affiliations:** 1 Cicely Saunders Institute of Palliative Care, Policy & Rehabilitation, King’s College London, London, UK; 2 Department of Surgery, Ophthalmology, Otorhinolaryngology and Physiotherapy, University of Valladolid, Soria, Spain; 3 Learning, Media and Inclusion (LMI), Department of Didactics and Educational Organization (DOE), Faculty of Education, University of Barcelona, Barcelona, Spain; 4 School of Dentistry, Cardiff University, Cardiff, UK; 5 Centre for Trials Research, Cardiff University, Cardiff, UK; 6 Department of Clinical Psychology and Psychological Therapies, Norwich Medical School, University of East Anglia, Norwich, UK; 7 Department of Epidemiology and Data Science, Amsterdam UMC, Vrije Universiteit Amsterdam, Amsterdam, Netherlands; 8 Amsterdam Public Health Research Institute, Methodology, Amsterdam, Netherlands

**Keywords:** Systematic reviews, Outcome measurement instruments, Content validity, Patient-reported outcome measures, Measurement properties, COSMIN

## Abstract

**Purpose:**

Systematic reviews of Patient-Reported Outcome Measures (PROMs) are essential for selecting appropriate measures for research and clinical practice. The COnsensus-based Standards for the selection of health Measurement INstruments (COSMIN) guidelines provide a standardised framework for evaluating measurement properties of PROMs, with content validity considered to be the most important property, yet the most complex and poorly reported. This study aimed to report common challenges researchers face when evaluating content validity in systematic reviews on the quality of PROMs, and to provide clear, experience-informed recommendations to overcome them.

**Method:**

A narrative synthesis of challenges in evaluating content validity in systematic reviews of PROMs is presented. Challenges were extracted from Elsman et al.’s umbrella review and from stakeholder consultations with the authors of this manuscript, providing practical recommendations and illustrative examples.

**Results:**

Seven challenges were identified: (1) feasibility of conducting a systematic review on the quality of PROMs, (2) identifying all articles describing PROM development for the target population, (3) managing different versions of PROMs, (4) poor reporting in primary studies (5) ambiguity in classifying studies as PROM development or content validity studies, (6) applying the criteria for content validity, specifically, to the results of development studies, and (7) accurately framing the scope of the PROM and the review. Recommendations include narrowing the scope of the review, conducting supplementary searches, treating PROM versions distinctly, and guidance on how to interpret poorly reported studies.

**Conclusion:**

These insights support consistent application of COSMIN guidelines and improve PROM selection and use, complementing existing COSMIN materials.

**Supplementary Information:**

The online version contains supplementary material available at 10.1007/s11136-026-04261-5.

## Introduction

A systematic review of the quality of Patient-Reported Outcome Measures (PROMs) can be conducted to support the selection of PROMs for use in clinical trials and clinical care [1]. PROMs are increasingly embedded in core outcome sets, recommended by regulators, and used to inform clinical decision-making and value-based healthcare. Ensuring PROMs are content-valid for the targeted construct and population is essential. A systematic review of PROMs aims to synthesise and compare the results of studies reporting measurement properties. Based on the available evidence and its quality, the most suitable PROM is then recommended for use. Systematic reviews of PROMs are complex and challenging, requiring the assessment of nine measurement properties [1]. Each of these properties present their own design requirements, which need to be understood by the review team, to conduct their systematic review [2].

The COnsensus-based Standards for the selection of health Measurement INstruments (COSMIN) guidelines were developed to support researchers and to standardise the process of conducting systematic reviews of the measurement properties of PROMs [1]. The guidelines were first published in 2010 as a risk of bias checklist [2]. Updates to the guidelines are based on additional Delphi studies with key stakeholders [3] and experiences of working with earlier versions of the COSMIN guidelines [4]. The current COSMIN guidelines were published in 2024 [1]. A PRISMA-COSMIN guideline was also developed to standardise the reporting of systematic reviews of PROMs [5, 6].

Within these guidelines, content validity is defined as the degree to which the content of a PROM is an adequate reflection of the construct to be assessed [3]. It is considered the most important measurement property [3], as without evidence of content validity, it may not be clear what the PROM is measuring, and therefore wrong conclusions may be drawn about the outcome that is intended to be measured [3].

Content validity comprises three aspects: 1) relevance (the degree to which the content of a PROM is an appropriate reflection of the construct of interest for the target population and its applied context), 2) comprehensiveness (no key aspects of the construct are missing) and 3) comprehensibility (individuals completing the PROM understand the instructions, items and response options as intended) [7].

Although content validity is the most important measurement property, it is also the most complex to evaluate. Often, content validity is poorly reported (if at all) in PROM development and validation articles, meaning there is no evidence to support this measurement property [8]. Poor or incomplete assessment of content validity carries important consequences. PROMs that fail to fully capture the construct of interest or include irrelevant, unclear, or incomplete items may lead to misinterpretation of trial outcomes, inappropriate decisions about treatment effectiveness, and inefficient allocation of resources [9, 10]. Ultimately, this can result in participant or patient burden without benefit.

When conducting a systematic review of PROMs, review teams can use the following sources to evaluate content validity: 1) the original development study that includes details of concept elicitation and pilot testing with the target population; 2) additional content validity studies (if applicable); 3) the review team should rate the three aspects of content validity (i.e., relevance, comprehensiveness and comprehensibility) of the finalised measure themselves, if a copy of the PROM is available [1].

Each month, COSMIN hosts international research clubs for researchers with a shared interest in health-related measurement, systematic reviews of PROMs and clinimetrics. During Club meetings, researchers have expressed numerous challenges when assessing the content validity of PROMs whilst conducting their own systematic reviews. The purpose of this article is to report common challenges researchers face when evaluating content validity in systematic reviews on the quality of PROMs and to provide clear, experience-informed recommendations to overcome them. By strengthening the assessment of content validity, we aim to support better PROM selection, more reliable research conclusions, and improved patient-centred outcomes.

## Methods

We present a narrative synthesis of challenges experienced by researchers conducting systematic reviews on the quality of PROMs.

First, we identified challenges reported in systematic reviews of PROMs by drawing on a published umbrella review. In 2024, Elsman et al. [8] conducted an umbrella review of the methodological quality of 100 systematic reviews of health-related outcome measurement instruments, randomly selected from the COSMIN database of systematic reviews (https://www.cosmin.nl/tools/database-systematic-reviews/). Full methods are reported elsewhere [8]. From this umbrella review, we selected reviews that reported on content validity. Each article was reviewed by a member of the review team (RLC, LBM, RP, or SLM), and data were extracted on (1) challenges or difficulties in reviewing or rating content validity in primary studies and (2) author-reported solutions. Systematic reviews were not identified through any other searches.

Secondly, we held an online stakeholder consultation meeting, which included all authors of this manuscript. The team comprises seven researchers, including two PhD students, two post-doctoral researchers, two lecturers and a professional clinical psychologist (all authors). The group have experience co-hosting (RLC, SLM, LM), attending COSMIN-European research clubs (AG, RP, TP, JH), or developing COSMIN guidelines (LM). All authors have conducted their own systematic reviews on the quality of PROMs using the COSMIN guidelines [11–15]. During the consultations, the team was asked to share their own experiences of conducting systematic reviews, the challenges they faced in relation to reviewing or rating content validity, and solutions they used to overcome them. Notes were made during the meeting.

We then met as a study team to discuss and synthesise the identified challenges from included studies and stakeholder consultations. Together we agreed to present the challenges in the order they would typically arise when following the COSMIN guidelines. We then developed recommendations and solutions on how to overcome them, based on COSMIN guidelines for conducting systematic reviews of PROMs version 2.0 [1]. These were supplemented with examples from the team’s own experiences. The final two challenges were suggested by LM as developer of the COSMIN guidelines, with the agreement of co-authors.

## Results

From Elsman et al.’s [8] umbrella review we identified sixteen papers reporting on five different challenges when evaluating content validity. Author-reported solutions were only included in three papers [16–18]. The aim of the included systematic reviews and author-reported challenges and solutions when assessing content validity are shown in Table 1 of Supplementary File 1.

**Table 1:** Examples illustrating how to apply the standards in the risk of bias checklist

Example 1. Quote from Imam et al. [29] included in Lahuerta-Martín et al.’s [13] systematic review
“Based on our clinical experience and feedback from individuals with Spinal Cord Injury (SCI) we modified the MFIS (MFIS-SCI–Appendix A) because not all the items were deemed relevant. Three individuals with SCI and two physiatrists with SCI experience reviewed the content, response categories and language of the items. The response range of the scale was changed from ‘past 4 weeks’ to ‘past week’ because the medical status of patients with SCI may change significantly in 4 weeks compared with 1 week. Three items (4, 13 and 20) from the original scale were deleted because the content did not reflect the SCI experience. For example, item 13 ‘my muscles have felt weak’ was deleted. Three new items (5, 15 and 19) were added as they more closely represent the SCI experience. Phrases ‘away from home’ and ‘at home or at work’ were removed from items 9 and 16 of the original scale, respectively, to generalize the items to a wider spectrum of SCI patients who are discharged from rehabilitation into the community or into an assisted care facility”.
Recommended risk of bias rating: inadequate *Explanation:* Although the authors evaluated relevance, comprehensibility (‘language of the items’) and comprehensiveness (by adding new items as the original PROM was insufficient), they stated that modifications were introduced based on their own experience and feedback from three patients and two healthcare professionals. As no details were described about how this feedback was collected, it suggests that no appropriate method of data collection was used (Box 1, standard 2). Therefore, an inadequate rating was given.

Example 2. Quote from Palimaru et al. [20] included in Lahuerta-Martín et al.’s [13] systematic review
“We conducted 20 in-depth interviews with adults with SCI who are full-time wheelchair users, exploring quality of life in the context of SCI, and preferences for different health outcome measures, with findings reported elsewhere. The need for 2 separate scales emerged from these in-depth interviews. […]. Participants disclosed that, for many activities, physical and mental fatigue can compete and conspire to frustrate and reduce what a disabled person can do.”
Recommended risk of bias rating: Doubtful *Explanation:* The authors explicitly state within the manuscript that in-depth interviews were held (Box 1, standard 2 was rated as ‘very good’). However, no more details were provided, e.g. about the skills of the interviewer (standard 3) or the use of a topic guide (standards 4). Therefore, the review team did not know what exactly was done. Therefore a ‘doubtful’ rating was given

Example 3. Quote from Wood et al. [30] included in Greco et al.’s [14] systematic review
“Following development by the authors, the child version of RSES (CRSES) scale was reviewed by two experts. Both experts had extensive experience in using the original scale as well as expertise in questionnaire development and validation. The experts were asked to independently examine the extent to which the items on CRSES could be understood by children and assessed their SE. The suggestions from experts were then incorporated into the updated scale. The main suggestion from the reviewers was to alter the response options; these were amended from “strongly agree,” “agree,” “disagree,” and “strongly disagree”; to “very true,” “true,” “not true,” and “definitely not true.”
Recommended risk of bias rating: doubtful; recommended rating for good content validity: Indeterminate *Explanation:* The results are poorly reported, since there is no information about the relevance, comprehensiveness or comprehensibility of the items within the manuscript, the review team rated the risk of bias as ‘doubtful’. Subsequently, an ‘indeterminate’ rating was given for all three aspects of content validity, given that not enough information was reported.

During the stakeholder consultations with members of the review team, we identified five challenges and developed solutions for each challenge, which are presented in Table 2 of Supplementary file 1. Some of these challenges overlapped with those identified from Elsman’s umbrella review.

The review team met to synthesise the findings, which were subsequently summarised into seven challenges. These are outlined below and presented in the order by which a review team would conduct a systematic review and assess content validity in accordance with COSMIN guidelines [1]. We summarise each challenge, followed by recommendations and at least one example.

### Challenge 1: Feasibility of conducting a systematic review on the quality of PROMs

A systematic review of PROMs can be time consuming. Searches may reveal a large volume of literature that needs to be assessed for eligibility. The searches may identify too many articles, or too many PROMs, making the review unfeasible, particularly if the review team have limited resource (e.g., time, small research team, etc.). To resolve this issue, some reviewers choose not to critically assess content validity as it was perceived to be too time consuming.

#### Recommendation

Content validity is the most important measurement property. It needs to be clear that the items of the PROM are relevant, comprehensive, and comprehensible to the construct of interest and target population. Poor content validity can affect all other measurement properties [2].

The review team should assess the feasibility of their review by running preliminary searches to understand whether the volume of literature identified can be adequately reviewed using the resources available.

To make the review feasible, an alternative solution may be to conduct a systematic review that solely focuses on reviewing the content validity of identified PROMs. The remaining eight measurement properties may then be addressed in a separate review. If the review team finds high-quality evidence for insufficient content validity, those PROMs can be excluded from subsequent reviews.

#### Examples

In a recent COSMIN systematic review by Hassan et al. [15], the authors identified more than 30 instruments intended to measure wisdom. To enhance feasibility, the review team chose to narrow the scope by including only self-report measures, thereby excluding performance-based instruments.

When reviewing instruments to assess child maltreatment through parents or caregivers, Yoon and colleagues [19] analysed content validity separately from internal consistency, reliability, measurement error, structural validity, hypothesis testing, cross-cultural validity, and criterion validity.

### Challenge 2: Identifying all articles describing PROM development for the target population

The original development study (i.e., describing the concept elicitation or pilot study) may not be identified through initial database searches. Generic PROMs may have been developed for a different population and may not meet the review’s eligibility criteria. Sometimes the development process (e.g., for older instruments) may not be published in peer-reviewed journals. Instead, they may be reported in user manuals or other grey literature not captured by traditional literature databases searches.

#### Recommendation

If information about the development of an included PROM is lacking from the initial literature search, the review team will need to conduct additional searches (e.g., through reference checking of included articles, additional searches of the (grey) literature, or contacting the authors directly) to identify this information. Manuals may be acquired using generic search engines, or PROQOLID (a research instrument database).

If the required information cannot be found in additional resources, the review team may decide to exclude the PROM from further evaluation.

#### Example

Lahuerta-Martín et al. [13] conducted a systematic review evaluating the measurement properties of PROMs measuring fatigue in people with spinal cord injury. Their search strategy identified several articles describing fatigue PROMs, including the Fatigue Severity Scale [20]. The Fatigue Severity Scale was originally developed for people living with multiple sclerosis and lupus erythematosus. The original development article was identified through additional searches of the literature using the PROM name. The original development study was rated using the COSMIN Risk of bias checklist, and its results were applied against the criteria for good content validity (see manual Table 6.6 [1]).

### Challenge 3: Managing different versions of PROMs

There may be different versions of the same PROM. This may be due to 1) adaptations/modifications to an existing measure (e. g. adding or removing items, changing the response scale); 2) condensing the original PROM into a short form; 3) translating the PROM into different languages, etc. Content validity (and other measurement properties) may be rated differently for each version of the PROM. With multiple articles describing the quality of different versions of the PROM, it may be challenging to decide which study results to summarise within the systematic reviews on the quality of PROMs.

#### Recommendation

In general, the COSMIN guidelines consider each version of a PROM to be unique [1]. Each aspect of content validity (relevance, comprehensiveness and comprehensibility) should be assessed and summarised separately for each version of the PROM. Results should not be combined into a single summary across versions unless there is evidence that the versions can be treated as equivalent. This may be the case when: 1) item functioning does not differ between versions (e.g., translated versions), or 2) reliability studies show no systematic difference between versions (e.g., paper-and-pencil vs. computer-based administration). Authors of the review should clearly state which populations were used to assess content validity.

A special case would be when a short form of a broader item bank is included in a systematic review. In such cases, results about relevance and comprehensibility of the development of the whole item bank can be used to draw conclusions on the relevance and comprehensibility of the short form. However, results on comprehensiveness of the item bank may not be generalized from the short forms, as only a subset of items are included. Conclusions on comprehensiveness may only be based on the reviewer’s ratings, when no other studies are available.

#### Example

In their systematic review, Lahuerta-Martín et al. [13] identified two short forms—PROMIS Fatigue Short Forms 7a and 8a—that measure fatigue from the broader PROMIS Fatigue item bank. The authors treated each short version as a distinct PROM, in line with COSMIN guidelines. When evaluating content validity, they searched for the original PROM development studies for the PROMIS Fatigue item bank. Each article was rated using: 1) the COSMIN Risk of bias checklist (to assess the methodological quality of the study), and 2) the COSMIN criteria for good content validity (manual Table 6.6 [1]). They used the results of relevance and comprehensibility in the evaluation of the short forms. However, the results of comprehensiveness were not used, and the comprehensiveness of both short forms were rated as indeterminate. As there were no additional content validity studies, only the development studies of the whole item bank and reviewer team ratings were used to draw conclusions for each aspect of content validity for each of the two short forms.

### Challenge 4: Poor reporting in primary studies

Review teams have reported difficulties assessing the quality of development studies or studies on content validity due to a lack of information or poor reporting in primary studies [18, 21–28].

#### Recommendation

When studies are poorly reported, the standards in the COSMIN risk of bias checklist suggest the study should be rated as ‘doubtful’, as it is unclear what was done. When the authors explicitly state that something was not done, or it is unlikely (e.g., interviews were not recorded or transcribed verbatim, box 1, standard 5 [1]) the standard should be rated as ‘inadequate’. These ‘doubtful’ ratings can influence the conclusions drawn about the content validity. For example, if standard 8 in box 1a (i.e. was data collection continued until saturation was reached?) is not rated as ‘very good’ or ‘adequate’, criteria 6 (i.e. whether saturation was reached) is not clear, which lead to an ‘indeterminate’ rating about the content validity of the PROM (see Table 1).

**Fig. 1:**
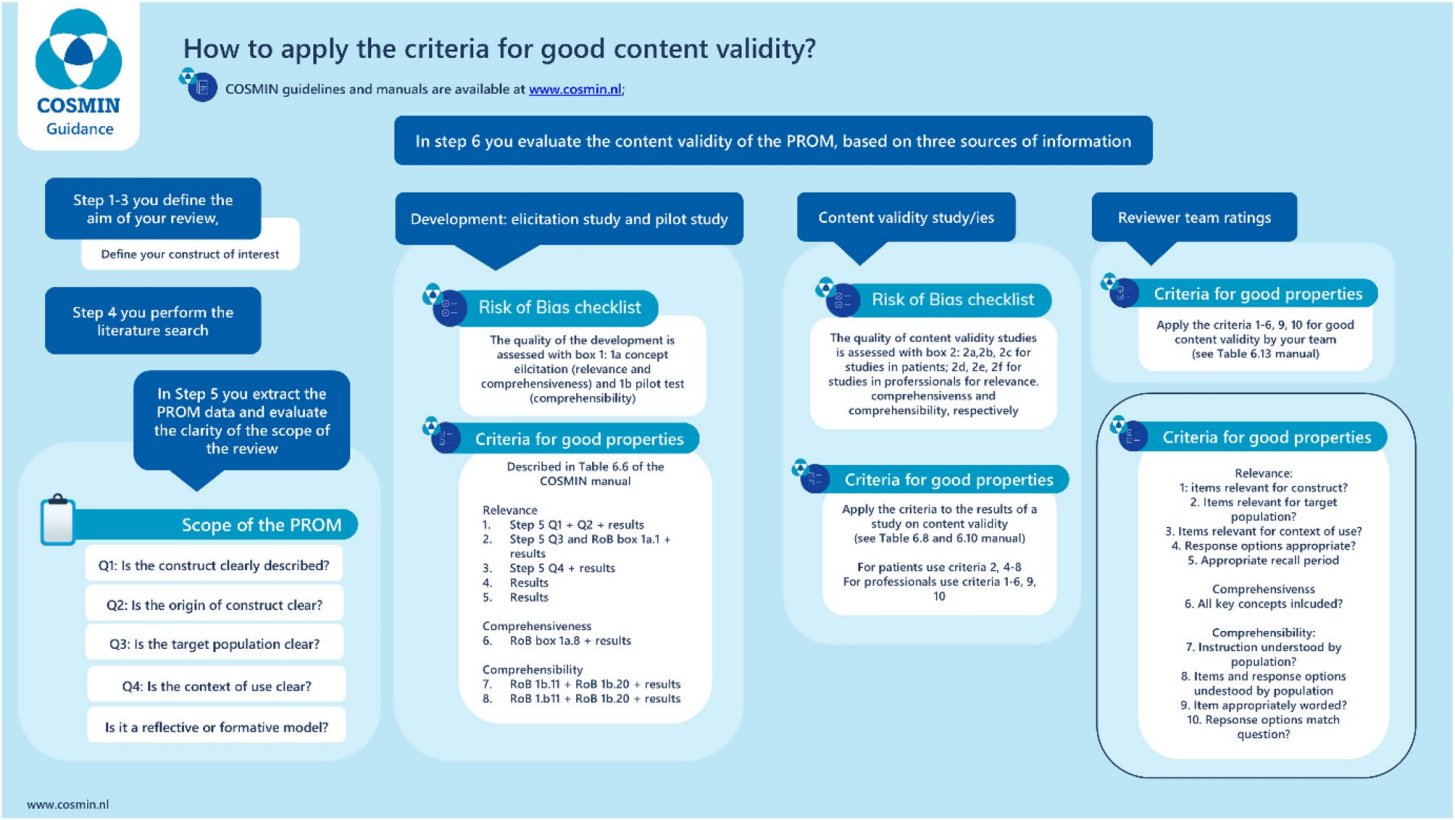
An outline of the information needed to apply the criteria for good content validity from development studies, additional content validity studies and the reviewer team ratings

If the review team have access to the PROM, they should evaluate content validity themselves (see Fig. 1), although it may result in ‘very low-quality evidence’ for content validity. To improve future reporting practices, in line with PRISMA-COSMIN reporting guidelines (Item 30b) [5] reviewers are encouraged to discuss limitations of included studies, including poor reporting and how future studies can address these gaps.

#### Example

Arts et al. [21] conducted a systematic review evaluating measurement properties of proxy-reported PROMs assessing physical activity, sedentary behaviour and/or sleep in children aged 0–5 years old. Studies included in their review had limited information on the methods used to develop their measure and, on the methods used to evaluate content validity. In the absence of this information, the evidence on content validity was rated as ‘doubtful’. In their discussion, the authors of the review highlighted these limitations (e.g. that key information was missing on whether interviewers were trained, whether data was coded independently, and whether data saturation was reached).

### Challenge 5: Ambiguity in classifying studies as PROM development or content validity studies

Sometimes, the review team may find it difficult to determine whether an article is reporting a PROM development study or a content validity study [8]. This may be particularly relevant if the content validity of an existing PROM is evaluated and it is concluded that it is insufficient, and proposals for a new version are presented within the article. In addition, it may be unclear whether an evaluation of comprehensibility in a new sample should be considered a pilot test within the development phase, or a content validity study.

#### Recommendation

In both scenarios, the study can be considered both: a development study of the new version of the PROM as results informed adjustments and results in a new version, and a content validity study of the existing version of the PROM as results conclude that the content validity was insufficient (as adjustments were required). We recommend choosing the rating procedure that best reflects how the authors of the original study used the results.

Note that this issue is likely to occur in studies focusing on comprehensibility. The COSMIN guidelines are developed in such a way, that using any of the two procedures will lead to the same conclusion, since the aim is to collect and summarise evidence on content validity. For example, the standards included in box 1b for comprehensibility in the development study, and in box 2c for comprehensibility in a content validity study are the same, and the quality of a pilot study (investigating comprehensibility) is equally treated as the quality of a content validity study on comprehensibility in the certainty assessment (see manual Table 6.3) [1].

#### Example

In their systematic review, Lahuerta-Martín et al. [13] included the Fatigability Index [20]. This PROM was recently developed, and in the article several studies were described, both involving patients as well as studies involving professionals. The study involving patients conducted by the developers of the PROM, was considered to be a development study and was rated using boxes 1a and 1b of the COSMIN Risk of Bias checklist, and Table 6.6 of the manual [1]. Part of the development involved professionals. To evaluate the study involving professionals two strategies could be used: 1) to consider it as a content validity study and use boxes 2d–2f [1]; or 2) to ignore the study, as there were no standards for evaluating risk of bias of development studies involving professionals.

### Challenge 6: Applying the criteria for content validity, specifically to the results of a development study

There are 10 criteria for good content validity: five for relevance, one for comprehensiveness, and four for comprehensibility.

#### Recommendation

When evaluating each of the different measurement properties in a systematic review using the COSMIN guidelines, *study quality* is rated using the risk of bias checklist, and the *PROM quality* is rated using the criteria for good measurement properties. Applying the criteria for good content validity is slightly different compared to other measurement properties. This is because the results of a content validity study are the PROM itself. The review team may find quantitative or qualitative evidence in the article, which we recommend highlighting in the review, rather than extracting into a database. To apply the criteria to development studies, the review team need the results of the ratings in step 5 about the clarity of the construct, target population and context of use, as well as the risk of bias ratings from step 6 [1].

Moreover, the set of relevant criteria differs for development studies (manual Table 6.6 [1]), content validity studies (manual Table 6.8 and 6.10 [1]), and the reviewer team ratings (manual Table 6.13 [1]).

#### Example

Figure 1 provides an outline of the information needed to apply the criteria for good content validity from development studies, additional content validity studies and the reviewer team ratings.

### Challenge 7: Scope of the PROM

The scope of the PROM refers to the target construct and the target population for which it was developed, and the intended context of use (manual p34–5 [1]). This scope of the PROM may differ to the scope of the review. Depending on the clarity of these descriptions (construct, population and context) challenges may occur: e.g. a) What to do if some of these aspects are not clearly described? and b) what to do when the aspects are clearly described, but the scope of the study is different from the scope of the review?

#### Recommendation

In step 5 of conducting a systematic review, the review team should extract the scope of the PROM, as described by the developers of the PROM. The review team should also rate (yes/no) whether the construct was clearly defined (Q1), whether the origin of the construct (Q2) and target population were clear (Q3), and whether the context of use was clearly described (Q4).

(A) When these aspects are not clearly described, some of the criteria will be rated as ‘indeterminate’. For example, criteria 1 for the development study asks whether 85% of the items refer to the construct of interest (manual Table 6.6). If the construct of interest is not clearly described, the criterion is assigned an ‘indeterminate’ rating [1].
(B) When these aspects are clearly described, the criteria should be rated as the results of the studies, even when PROM and review may not share the scope. I.e., if Q1-Q4 are clearly stated and the results are ‘sufficient’, the criteria are rated as ‘sufficient’, even if the scope of the study is different to that of the review. However, in the grading phase, where all evidence is considered, the review team may decide to downgrade the evidence for content validity of the PROM for indirectness (manual p158 [1]).

#### Example

The aim of the systematic review by Lahuerta-Martín et al. (2025) [13] was to evaluate the quality of PROMs assessing fatigue in patients with spinal cord injury. One of the included scales was the PROMIS Fatigue Short Form (SF) 8a. This scale was originally developed for the general population. The content validity for this scale was based on two development studies (both evaluated with the general population). Both studies were rated as having sufficient content validity with adequate study quality. No additional studies on content validity were found. The review team rated the PROMIS Fatigue SF 8a themselves using Table 6.13 of the manual. They agreed to rate the PROM as ‘sufficient’. To grade the evidence, Table 6.3 of the manual was used. As the results were consistent, but the target population of the development study differed from that of the systematic review, the review team decided to start with ‘high-quality evidence’ (i.e. two studies of adequate quality), and downgrade by one level for indirectness, resulting in ‘moderate evidence for sufficient content validity’.

## Discussion

The evaluation of content validity in a systematic review of PROMs is an important and often challenging step to take. This article can act as an aid for review teams conducting systematic reviews of PROMs. The recommendations and examples provide clear illustrations of how to apply the COSMIN criteria [1]. These were derived from the COSMIN guidelines, an umbrella review of COSMIN-based systematic reviews, and the shared experiences of the authors whilst conducting their own reviews. By sharing our learnings, we hope this will support the assessment of content validity in future systematic reviews of the quality of PROMs.

Content validity is considered the most important measurement property. Without sufficient content validity, it may not be clear what the PROM is measuring, and wrong conclusions may be drawn about an outcome that is measured. If there is high-quality evidence that content validity is insufficient, the PROM should only be furtherly assessed or recommended for use on the basis that it evidently requires improvements. Results on the different components of content validity can be used to provide recommendations for such improvements.

We identified seven key challenges, but additional challenges may exist. The COSMIN methodology is developed as a systematic approach to synthesise evidence on the quality of PROMs. This article is intended as a supplement to the COSMIN guidelines: researchers can use this paper to educate themselves on appropriate application of the COSMIN guidelines and to find solutions when uncertainties arise regarding content validity assessment. With the expanded use of COSMIN guidelines and the increasing interest in conducting systematic reviews of PROM measurement properties [7], we expect new challenges will arise, not only related to the assessment of content validity, but additional measurement properties. COSMIN materials and research clubs will continue to grow and adapt based on the scientific community’s feedback and experience, to provide useful tools and guidance. The explanation of these challenges, and recommended solutions will support the consistency with which COSMIN guidelines are applied when conducting systematic reviews of PROMs.

Patient and public partners were not involved in this study. Given that the paper focuses on methodological guidance for applying COSMIN guidelines, this topic was considered unlikely to meaningfully benefit from patient or public partner involvement.

## Supplementary Information

Below is the link to the electronic supplementary material.

Supplementary file1 (DOCX 26 KB)

## Data Availability

No datasets were generated or analysed during the current study.
