# Supplementary material for: Assessing content validity: challenges of conducting systematic reviews of patient-reported outcome measures and recommendations to improve the application of COSMIN guidance
Source: Qual Life Res. 2026 May 3;35(6):157. doi: 10.1007/s11136-026-04261-5 (PMC13136209; doi:10.1007/s11136-026-04261-5)
Supplement: Supplementary file 1 — Supplementary file1 (DOCX 26 KB) [file 11136_2026_4261_MOESM1_ESM.docx]

**Supplementary File**

*Table 1. Characteristics of included systematic reviews and author-reported challenges and solutions when assessing content validity.*

| **Authors** | **Study aim** | **Author-reported challenges assessing content validity** | **Author-reported solutions** |
| --- | --- | --- | --- |
| Alaqueel et al. [1] | To identify and summarize and synthesize the studies reporting the development, translation, and/or psychometric evaluation of PROMs in Arabic-speaking populations  To evaluate the psychometric properties and the translation process of included PROMs | Lack of available studies reporting the development process | Not reported |
| Arts et al. [2] | To summarise available studies evaluating measurement properties of proxy-report questionnaires assessing physical activity, sedentary behaviour and/or sleep in children aged 0–5 years | Studies do not address all aspects of content validity (comprehensiveness, relevance and comprehension) | Not reported |
|  |  | Methods poorly reported | Not reported |
| Baqays et al. [3] | To perform a comprehensive  systematic review of the available literature on patient reported outcome questionnaires that assess swallowing dysfunction in otherwise healthy infants and toddlers | Results poorly reported | Not reported |
| Essiet et al. [4] | To critically evaluate the psychometric properties of teacher proxy-report instruments for assessing one or more of the 30 elements within the four domains of the Australian Physical Literacy Framework (APLF), in children aged 5–12 years  To examine the alignment of each tool (and relevant items within) with the APLF and provide recommendations for teachers in assessing physical literacy in children aged 5–12 years | Methods poorly reported | Not reported |
|  |  | Poor methodology (e.g. no pilot tests or cognitive interviews) | Not reported |
| Fan et al. [5] | To critically evaluate the Patient-Reported Outcome Measures for masticatory function in adults | Methods poorly reported | Not reported |
| Le et al. [6] | To identify the most suitable existing generic and condition-specific health-related quality of life patient-reported outcome measures for active youth with and without a musculoskeletal injury, based on measurement properties, interpretability and feasibility | Studies do not address all aspects of content validity (comprehensiveness, relevance and comprehension) | Not reported |
|  |  | Methods poorly reported (e.g. unclear if patients were asked about comprehensiveness and comprehension) | Not reported |
| Lear et al. [7] | To systematically review self‐reported measures of generalised shame | Conflicting findings in multiple papers assessing the content validity of a PROM | Applied inconsistency ratings to subdomains of content validity (relevance, comprehensiveness and comprehensibility) to be specific about what domain was affected by conflicted findings |
|  |  | Lack of available studies reporting the development process and assessment of content validity | Not reported. |
|  |  | Poor methodological quality (small sample sizes) | Apply an indeterminate or inconsistent rating where methods are poor or poorly reported |
|  |  | Poor reporting of methods | Apply an indeterminate or inconsistent rating where methods are poor or poorly reported |
| Lodge et al. [8] | To appraise the measurement properties of generic patient-reported outcome measures (PROMs) measuring postoperative quality of life in adults undergoing elective abdominal surgery | Unable to identify additional studies on content validity | Not reported |
| Luck-Sikorski et al. [9] | To aggregate and evaluate evidence of psychometric properties and methodological quality of published measures to assess stigma in visible skin diseases | Methods poorly reported | Not reported |
| Marisco et al. [10] | To systematically review the psychometric properties and feasibility of outcome measures assessing lower limb somatosensory function and body awareness in children with upper motor neuron lesion | Lack of information on content validity | Not reported |
| Phillips et al. [11] | To examine the validity, reliability and feasibility of tools used to assess sleep of pre-school aged children | Methods poorly reported | Not reported |
| Ratti et al. [12] | To systematically review the measurement properties of patient-reported outcome measures (PROMs) used in localised prostate cancer and recommend PROMs for use in routine practice and research settings | Difficulties assessing content validity when multiple content validity papers are identified | Subjective judgement by the review team to assess the adequacy of the methods used |
| Roos et al. [13] | To systematically review patient-reported outcome measures (PROMs) of musicians' musculoskeletal symptoms (MSS) and psychosocial factors and their psychometric properties | Methods poorly reported | “Doubtful” scores are given when parts of the methodology are unknown, while “adequate” is given when methods are reported but deemed inadequate |
|  |  | No PROM development studies published | Not reported |
| Theon et al. [14] | To identify self-report measures that have been used in populations on the autism spectrum with regard to reporting stress  To identify whether the information regarding the psychometric properties of these tools is present for individuals on the autism spectrum | No information on content validity | Not reported |
| van Raath et al. [15] | To appraise the content validity and other measurement properties of outcome measurement instruments for port wine stain treatment to identify the most appropriate instruments and future research priorities | No PROM development studies published | Not reported |
|  |  | Substandard methods to assess content validity (e.g. no reports of pilot testing or asking professionals about comprehensiveness etc.) | Not reported |
| Wang et al. [16] | To identify patient-reported outcome measures (PROMs) for intermittent self-catheterization (ISC) users, critically assess and summarize the quality of the measurement properties, and describe the application scenarios on each instrument. | Studies do not address all aspects of content validity (comprehensiveness, relevance and comprehension) | Not reported |

*Table 2. Challenges and solutions of assessing content validity, identified through stakeholder consultations*

| **Challenges assessing content validity** | **Solutions** |
| --- | --- |
| Searches identifying too many papers, or too many PROMs, making the review unfeasible | Conduct a systematic review that solely focus on reviewing the content validity of identified PROMs. The remaining eight measurement properties may then be addressed in a separate review |
| Original development study is not identified by initial search strategy | Conduct additional searches to identify the development study |
| Multiple articles describing the quality of different versions of the PROM makes it challenging to decide which study results to summarise within the systematic review | Each aspect of content validity (relevance, comprehensiveness and comprehensibility) should be assessed and summarised separately for each version of the PROM |
| Difficulty determining whether an article is reporting a PROM development study or a content validity study | Return to the original paper to understand how the results of the study were used to understand if the article is a development or content validity study |
| Ambiguity when applying the criteria for good measurement properties | Not reported |

**References**

1. *Alaqeel, S., Alfakhri, A., Alkherb, Z., et al. (2022). Patient-reported outcome measures in Arabic-speaking populations: a systematic review. Quality of Life Research, 31(5), 1309-1320.*

2. *Arts, J., Gubbels, J. S., Verhoeff, A. P., et al. (2022). A systematic review of proxy-report questionnaires assessing physical activity, sedentary behavior and/or sleep in young children (aged 0–5 years). International Journal of Behavioral Nutrition and Physical Activity, 19(1), 18.*

3. *Baqays, A., Zenke, J., Campbell, S., et al. (2021). Systematic review of validated parent-reported questionnaires assessing swallowing dysfunction in otherwise healthy infants and toddlers. Journal of Otolaryngology-Head & Neck Surgery, 50(1), 68.*

4. *Essiet, I. A., Lander, N. J., Salmon, J., et al. (2021). A systematic review of tools designed for teacher proxy-report of children’s physical literacy or constituting elements. International Journal of Behavioral Nutrition and Physical Activity, 18(1), 131.*

5. *Fan, Y., Shu, X., Leung, K. C. M., et al. (2021). Patient-reported outcome measures for masticatory function in adults: a systematic review. BMC oral health, 21(1), 603.*

6. *Le, C. Y., Truong, L. K., Holt, C. J., et al. (2021). Searching for the holy grail: A systematic review of health-related quality of life measures for active youth. journal of orthopaedic & sports physical therapy, 51(10), 478-491.*

7. *Lear, M.K., Lee, E.B., Smith, S.M., et al. (2022). A systematic review of self‐report measures of generalized shame. Journal of Clinical Psychology, 78(7), 1288-1330.*

8. *Lodge, M. E., Moran, C., Sutton, A. D., et al. (2022). Patient-reported outcome measures to evaluate postoperative quality of life in patients undergoing elective abdominal surgery: a systematic review. Quality of Life Research, 31(8), 2267-2279.*

9. *Luck‐Sikorski, C., Roßmann, P., Topp, J., et al. (2022). Assessment of stigma related to visible skin diseases: a systematic review and evaluation of patient‐reported outcome measures. Journal of the European Academy of Dermatology and Venereology, 36(4), 499-525.*

10. *Marsico, P., Meier, L., van der Linden, M. L., et al. (2022). Psychometric properties of lower limb somatosensory function and body awareness outcome measures in children with upper motor neuron lesions: a systematic review. Developmental Neurorehabilitation, 25(5), 314-327.*

11. *Phillips, S. M., Summerbell, C., Ball, H. L., et al. (2021). The validity, reliability, and feasibility of measurement tools used to assess sleep of pre-school aged children: A systematic rapid review. Frontiers in Pediatrics, 9, 770262.*

12. *Ratti, M. M., Gandaglia, G., Alleva, E., et al. (2022). Standardising the assessment of patient-reported outcome measures in localised prostate cancer. A systematic review. European urology oncology, 5(2), 153-163.*

13. *Roos, M., Dagenais, M., Pflieger, S., et al. (2022). Patient-reported outcome measures of musculoskeletal symptoms and psychosocial factors in musicians: a systematic review of psychometric properties. Quality of Life Research, 31(9), 2547-2566.*

14. *Thoen, A., Steyaert, J., Alaerts, K., et al. (2023). A systematic review of self-reported stress questionnaires in people on the autism spectrum. Review journal of autism and developmental disorders, 10(2), 295-318.*

15. *van Raath, M. I., Chohan, S., Wolkerstorfer, A., et al. (2021). Treatment outcome measurement instruments for port wine stains: a systematic review of their measurement properties. Dermatology, 237(3), 416-432.*

16. *Wang, X., Cao, X., Li, J., et al. (2021). Evaluation of patient-reported outcome measures in intermittent self-catheterization users: A systematic review. Archives of Physical Medicine and Rehabilitation, 102(11), 2239-2246.*
